# Temporal discounting when outcomes are experienced in the moment: Validation of a novel paradigm and comparison with a classic hypothetical intertemporal choice task

**DOI:** 10.1371/journal.pone.0251480

**Published:** 2021-05-14

**Authors:** Virginie M. Patt, Renee Hunsberger, Dominoe A. Jones, Margaret M. Keane, Mieke Verfaellie

**Affiliations:** 1 Veterans Affairs Boston Healthcare System, Boston, MA, United States of America; 2 Department of Psychiatry, Boston University, Boston, MA, United States of America; 3 Department of Psychology, Wellesley College, Wellesley, MA, United States of America; McGill University, CANADA

## Abstract

When faced with intertemporal choices, people typically devalue rewards available in the future compared to rewards more immediately available, a phenomenon known as temporal discounting. Decisions involving intertemporal choices arise daily, with critical impact on health and financial wellbeing. Although many such decisions are “experiential” in that they involve delays and rewards that are experienced in real-time and can inform subsequent choices, most studies have focused on intertemporal choices with hypothetical outcomes (or outcomes delivered after all decisions are made). The present study focused on experiential intertemporal choices. First, a novel intertemporal choice task was developed and validated, using delays experienced in real time and artistic photographs as consumable perceptual rewards. Second, performance on the experiential task was compared to performance on a classic intertemporal choice task with hypothetical outcomes. Involvement of distinct processes across tasks was probed by examining differential relations to state and trait anxiety. A two-parameter logistic function framework was proposed to fit indifference point data. This approach accounts for individual variability not only in the delay at which an individual switches from choosing the delayed to more immediate option, but also in the slope of that switch. Fit results indicated that the experiential task elicited temporal discounting, with effective trade-off between delay and perceptual reward. Comparison with the hypothetical intertemporal choice task suggested distinct mechanisms: first, temporal discounting across the two tasks was not correlated; and second, state and trait anxiety both were associated with choice behavior in the experiential task, albeit in distinct ways, whereas neither was significantly associated with choice behavior in the hypothetical task. The engagement of different processes in the experiential compared to hypothetical task may align with neural evidence for the recruitment of the hippocampus in animal but not in classic human intertemporal choice studies.

## Introduction

Temporal discounting, or delay discounting, refers to the natural tendency to de-value rewards available in the future compared to rewards available more immediately in the context of intertemporal choice tasks [1–3]. Decisions involving intertemporal choices arise daily–e.g., deciding between spending money right away or investing it for later greater return; deciding between the immediate pleasure of caloric food consumption or healthier body sensations later. Whereas some degree of temporal discounting may be advantageous economically [4], excessive discounting has been related to a number of maladaptive behaviors, such as substance dependence [5], cigarette smoking [6], overeating [7], and non-adherence to medical treatment [8].

In light of the ubiquitous nature of intertemporal decisions, there has been much interest in identifying their cognitive and neural mechanisms. Animal models generally agree with brain imaging studies on the recruitment of a valuation network involving the ventral striatum and ventromedial prefrontal cortex [9–16]. Striking discrepancies, however, are reported across animal and human studies with regard to the recruitment of the medial temporal lobe. Specifically, whereas hippocampal lesions in humans do not appear to disrupt classic intertemporal decisions [17–19], animal studies have suggested a critical role for the hippocampus [20–26]. For example, rodents with surgical hippocampal lesions have been shown to be less willing to wait for greater later rewards compared to animals with sham lesions [20–22, 24]. These discrepancies are intriguing, as animal models are frequently used to establish the neural circuitry underlying human cognitive processes.

One key difference between human and animal tasks is that animals must *experience* the delays and consumption of rewards in real-time after they make their decisions (e.g., waiting 20 seconds to get 3 pellets of food). Reward values can therefore be revisited based on the experience of delays and rewards during the task. By contrast, most human research on the cognitive mechanisms underlying intertemporal choice has been conducted using questionnaire-type tasks, consisting of a series of hypothetical choices between a smaller reward available sooner and a greater reward available after various delays (e.g., “Would you prefer $90 now or $100 in a day?”, “in a month?”, “in a year?”) [27]. In such tasks, all decisions are made before consequences can be experienced, and decisions by definition must rely solely on semantic knowledge and reasoning. Intertemporal decisions may thus recruit different mechanisms depending on their “experiential” nature, that is whether they involve delays and rewards that are experienced in real-time and can inform subsequent choices [28].

The premise that different cognitive processes may be recruited depending on the experiential versus hypothetical nature of the task remains to be demonstrated in humans. Indeed, it is commonly assumed that temporal discounting across a variety of tasks in humans is supported by a common underlying mechanism [29], as evidenced by findings that intertemporal choices are reliable over time [30] and across tasks using different types of hypothetical rewards (e.g., money, drinks, food, cigarettes) [31–34] or delay temporal scales [35]. Studies that have directly compared participants’ choices with experiential and hypothetical outcomes are scarce and have reported mixed findings. Using as outcomes delivered in real-time depictions of numerical amounts of money or of coins piling up on a computer screen with later exchange for actual money, two studies found that such experiential decisions were significantly correlated with those in a hypothetical monetary task [36, 37]. By contrast, in another study that used monetary coins directly delivered to participants via a computer-connected slot machine, no correlation was observed with hypothetical decisions, suggesting that the tasks are mediated differently [38]. It is possible that a difference in the extent to which a reward can be directly experienced and elicit concomitant pleasure (i.e., reward consumability) contributed to these discrepant findings. The impact of reward consumability has been demonstrated in the hypothetical domain, with stronger correlations of discounting rates among tasks that used food rewards than across tasks comparing food and monetary rewards [33, 34, 39]. Similarly, in animal experiential tasks that used a secondary reinforcer that could be exchanged for access to food, delay to the exchange period was a more critical predictor of decisions than was delay to the presentation of the secondary reinforcer [40]. It is likely that reward consumability also has notable impact in human experiential tasks, as the experience of reward following an action is critical for updating reward values.

The present study aims to compare experiential and hypothetical intertemporal choice in humans by examining performance in a decision task with real-time delays and consumable rewards and in a classic task with hypothetical delays and rewards. We elicit temporal discounting in both cases using a simple binary decision format, involving a smaller reward delivered now and a fixed larger reward delivered after various delays (see [41] for other elicitation techniques). In the present work, we refer to tasks as hypothetical intertemporal choice tasks as long as all decisions are made before rewards are delivered. This conceptualization includes tasks that are incentivized, usually involving the payout of one randomly selected option among the participant’s decisions in the days to months after the experiment [27]. (We acknowledge that the term hypothetical is sometimes reserved for tasks in which participants expect no actual payout.) Based on minimal evidence of a systematic effect of monetary incentive on intertemporal choice [42–46], here we did not incentivize our hypothetical task with monetary payouts.

A number of experiential intertemporal choice tasks have been developed over the years, with delays spanning seconds to minutes and varying rewards, including U.S. dollar coins [38], visual or verbal representations of money on screens [37, 47–50], squirts of water or juice [51, 52], food [53, 54], pictures of socially attractive faces [55], sexually-arousing pictures [11], short cartoon videos [56], and video games [57, 58]. Arguably, the use of perceptual rewards is preferrable to the use of food or money: food may offer limited incentive for well-nourished participants and has been suggested to heavily depend on personal preferences [32]; and money, a secondary reinforcer, likely elicits a less direct experience of reward consumption in the moment. Tasks that have used audiovisual rewards all have quantified reward magnitude by modulating the duration of the reward (e.g., viewing a sexually arousing picture for 3 seconds after waiting 9 seconds or seeing it for 1 second after waiting 1.5 second [11]). Thus, these tasks could not evaluate the effects of temporal aspects of the delay independent of temporal aspects associated with the reward. In the present study, capitalizing on the rewarding effect of novel perceptual information [59, 60], we designed a novel experiential task that uses pleasant pictures as consumable reward; but instead of varying reward magnitude by manipulating its duration, we manipulated how much of the information contained in the pictures would be available through partial occlusion. Further, in contrast to previous studies that have used pictures that were rewarding only to a select group of individuals via displays of attractive faces of the opposite sex [55] or sexually-arousing pictures [11], we used artistic photographs that have general reward value (e.g., beautiful landscapes, wildlife, artistic architectures) and thus are suitable across various demographic groups.

The goals of the study were two-fold. First, we sought to validate the novel experiential task and propose a logistic function interpretive framework to examine temporal discounting. Second, we explored the mechanisms that may underlie experiential and hypothetical intertemporal choices by examining how performance on each of these tasks relates to individual differences in forms of anxiety that differentially tap in-the-moment processes.

To validate the novel experiential task as an intertemporal choice task, we demonstrate that viewing artistic photographs with varying levels of occlusion provides a continuum of perceptual reward and elicits a trade-off between delay and magnitude of perceptual reward (i.e., a temporal discounting effect). There is widespread recognition that temporal discounting can be modeled by a hyperbolic tradeoff pattern between delay and reward in hypothetical intertemporal choice tasks [1, 61]. Some studies, however, have suggested that a simple hyperbolic model may not provide optimal data fit [62, 63]. Here we demonstrate adequate hyperbolic fit to validate the experiential task, but also propose a logistic function framework for more precise and more intuitive modeling of temporal discounting.

**Fig 1:**
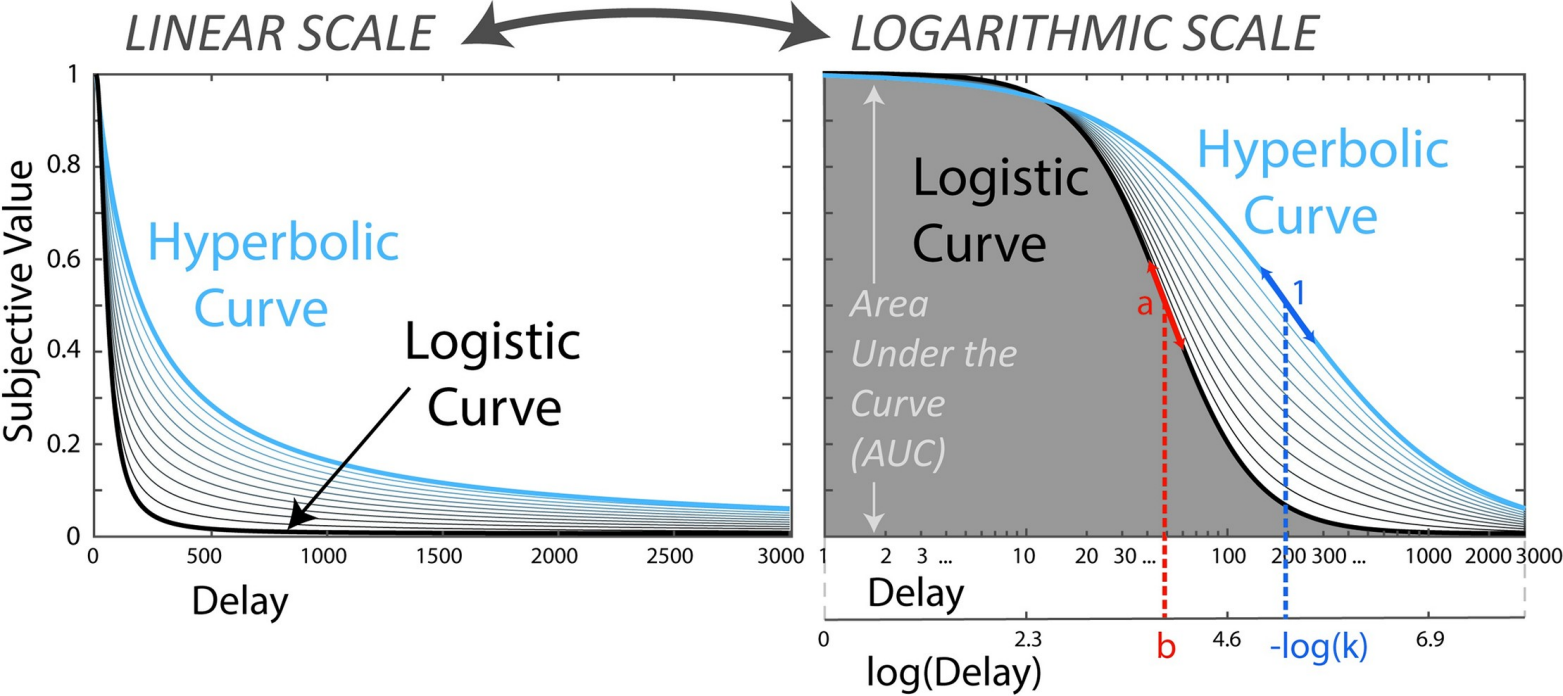
A logistic function framework to model indifference points data. Illustration of the transformation from a linear scale to a logarithmic scale of the classic hyperbolic curve; and illustration of the transformation from a logarithmic scale to a linear scale of a logistic function curve. The logistic curve is characterized by parameters *a* (the slope at the inflection point) and *b* (the log-delay at the inflection point). The hyperbolic curve is a particular type of logistic function with parameters *a = 1* and *b* = −log *k*, where *k* is the classic discounting rate.

The proposed logistic function interpretive framework was developed mathematically by noting that the hyperbolic model constitutes a subset of the logistic function [64] with logarithmic timescale and constant slope at the inflection point. Using a logarithmic time scale is inherent to most classic intertemporal choice tasks, with delays usually spanning weeks, months, and years that are grossly logarithmically distributed (e.g., 1 month, 2 months, 6 months, 1 year, 5 years, etc.). Such a distribution intuitively accounts for the Weber-Fechner law [65] that suggests diminished perception of change in a physical stimulus as the overall magnitude of that stimulus increases (e.g., one day may be perceived as substantial a week from now, but negligible in one year). In most intertemporal choice studies, however, whereas discounting rate parameters *k* are typically converted to log *k* for statistical analyses, indifference points are generally plotted using linear time scales. Such practice has obscured the underlying presence of an S-shape in the indifference curve, and has rendered the interpretation of log *k* less intuitive. With a logarithmic timescale, −log *k* can be interpreted as the log-delay at which the participant switches between consistently choosing the delayed option and consistently choosing the more immediate option (Fig 1).

In addition to making the logarithmic timescale more explicit, the logistic framework also allows for a flexible slope at the inflection point by incorporating an additional parameter, *a*. Although not within an explicit logistic function framework, a model has previously been proposed that is mathematically identical and has been shown to provide a better fit than the hyperbolic model [66, 67]. In this model, an exponent parameter *s*, mathematically equivalent to the logistic function parameter *a*, was applied to the delay. Letting parameter *a* (or *s*) vary has been suggested to account for individual variability in the subjective perception of time [66], akin to the increased flexibility of Stevens’ power law over the Weber-Fechner law for modeling subjective perception [68]. (See also [69] for another related model with the entire denominator exponentiated, and [63] for further demonstrations of superior fit of the model compared to classic models).

To explore the mechanisms underlying experiential and hypothetical intertemporal choices, we examined the relationship between temporal discounting rates and individual differences in state and trait anxiety. These two forms of anxiety relate to the same underlying construct yet differ in the extent to which they tap into in-the-moment processes. Moreover, they can be measured using a well-validated instrument and show considerable variability across a sample of college students, thus lending themselves well to an individual differences approach. We reasoned that state anxiety, reflecting anxiety in the moment, would be related more strongly to experiential than to hypothetical temporal discounting. This notion is supported by previous demonstrations of a selective effect of other manipulations of mental state on an experiential compared to a hypothetical task [70, 71]. Moreover, findings suggest that acute stress diminishes reward responsiveness in humans [72, 73] and blunts the effect of rewarding brain stimulation in rodents [74] under conditions where the reward is directly experienced. We therefore predicted that greater state anxiety would be associated with reduced sensitivity to different levels of experienced reward, resulting in choices driven more strongly by the experience of delay, and yielding increased temporal discounting (i.e., diminished willingness to wait). With regard to trait anxiety, which reflects a stable pattern throughout the lifetime, we predicted that, consistent with previous studies [[75–77] but see [78, 79]], it would be associated with greater temporal discounting in the hypothetical task. It was an open question whether the effects of trait anxiety would be dissociable across the hypothetical and experiential task. To foreshadow our findings, trait anxiety indeed had dissociable effects across the two tasks, albeit the association of trait anxiety with temporal discounting in the hypothetical task was not significant.

## Method

### Participants

Participants were 44 female college students with a mean age of 19.0 years (*SD* = 1.2) and mean education of 12.8 years (*SD* = 1.1), who indicated from self-report that they were free from major psychiatric disorder or neurological illness including attention deficit/hyperactive disorder (ADHD). This sample size was similar to previous studies that compared within-participant behavior across intertemporal choice tasks [29, 38, 46]. Depression levels of the sample (*M* = 13.6; *SD* = 7.9), as measured by the Center for Epidemiologic Studies Depression Scale, Revised [80], were similar to a normative sample of undergraduate students (*N* = 243, *M* = 16.4, *SD* = 13.5, *t(43)* = -1.9, *p* = 0.060, *Cohen’s d* = 0.25) [81]. The Wellesley College Institutional Review Board approved all experimental procedures, and all participants provided informed consent.

### Experimental tasks

#### Experiential intertemporal choice task

Capitalizing on the rewarding effect of novel perceptual information [59, 60], we used artistic photographs as consumable rewards. Reward value was manipulated by presenting the photographs with variable levels of occlusion in the form of lines overlaid on each photograph. The photographs (N = 200) were preselected from a bank of royalty-free pictures (www.pexels.com) to include only pleasant content (e.g., wildlife, beautiful landscapes, interesting architecture), with 85% of the photographs featuring primarily nature scenes. Because novelty was assumed to constitute a large part of the rewarding experience, photographs were drawn randomly from the bank of preselected photographs without repetition. Examination of photograph distribution across participants showed that they indeed viewed 85% of nature photographs on average (*SD* = 3%, *range* = [79%-91%], Shapiro-Wilk normality test: *W* = 0.980, *p* = 0.652), reflecting adequate functioning of the randomization algorithm and homogeneity in photograph content across participants.

During the task, participants had to decide between viewing a partially occluded photograph immediately or a non-occluded photograph after a delay. Immediate options comprised 10 possible occlusion levels and were constructed using varying line thickness and spacing that revealed 13%, 28%, 45%, 60%, 70%, 80%, 88%, 95%, 97% and 100% of the full photograph. Delayed options comprised delays of 1, 2, 4, 6, 8, 10, 12, 14, 16, 20, and 25 seconds.

For each participant, a series of choices was created using a semi-adaptive dichotomy algorithm, allowing efficient determination of indifference points at each preselected delay. Indifference points refer to the occlusion level for which viewing an occluded photograph now and viewing the full photograph after that delay have equivalent subjective value. For each preselected delay, the algorithm kept track of the uncertainty range for the indifference point. The uncertainty always ranged between 1 and 10 at the start of the task, corresponding to the ten available occlusion levels. On subsequent trials, the occlusion level of the immediate option was selected by each time cutting in half the uncertainty interval. For example, for decisions involving a delay of 10 seconds, the first trial involved viewing a photograph with occlusion level 5 now or a full view of that photograph after a 10 second delay. If the participant chose to wait, the uncertainty interval for a 10 second delay was reduced to between 1 and 5, and the next trial involved choosing between viewing a photograph with occlusion level 3 now or in full view after a 10 second delay. On each of the subsequent trials with a 10 sec delay, the uncertainty interval was again halved until it converged to zero, generally after 3 to 4 trials. In order to promote engagement with varied decisions, the occlusion level of the first trial, rather than starting systematically at level 5, was randomly chosen among levels 4, 5, and 6. Trials with different delays were interspersed, with random selection of delays for each trial among those that had not yet converged to an indifference point. The semi-adaptive algorithm was run twice yielding two indifference points per delay, for a total of 70 to 80 test trials per participant depending on the speed of convergence of the algorithm.

**Fig 2:**
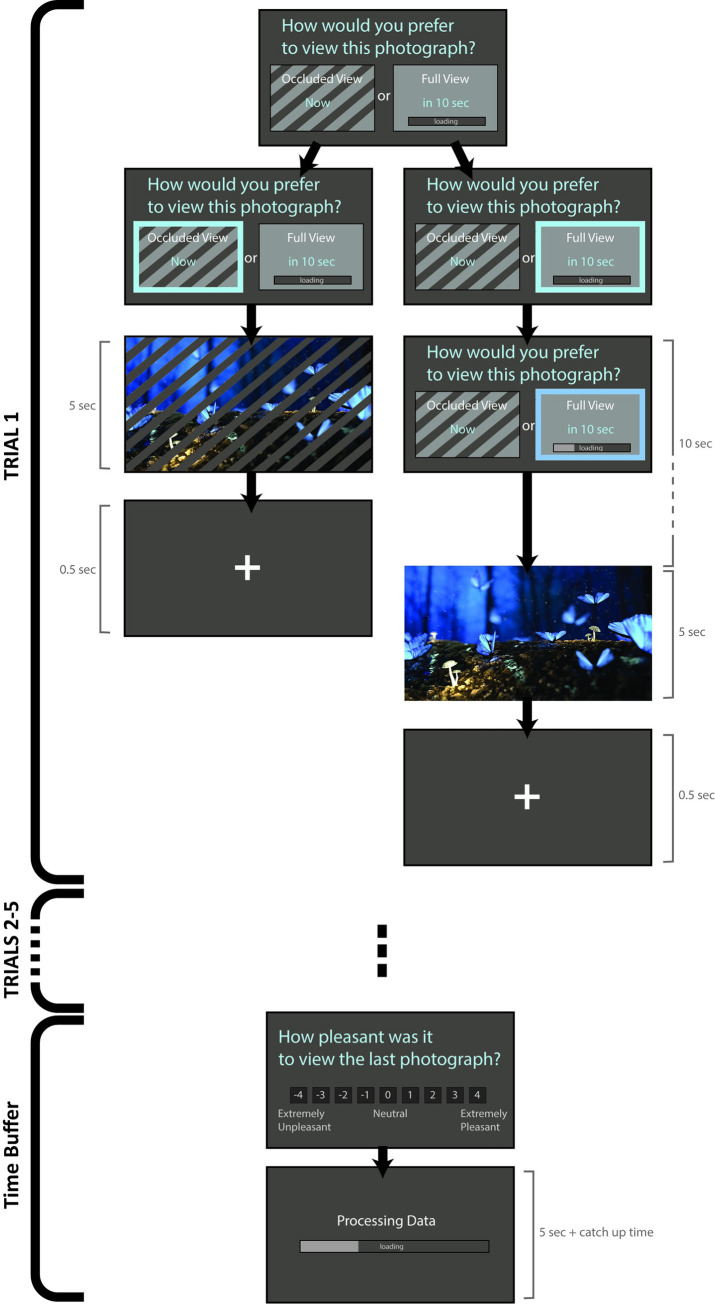
Experiential intertemporal choice task. The task required making a series of decisions between viewing a partially occluded photograph immediately or a non-occluded photograph after a delay. Outcomes unfolded in real-time following each decision. Every five trials, participants were asked about the pleasantness of their experience and waited through a “Processing Data” screen, with duration designed to equalize experimental time across participants.

On each trial, choices were represented visually on the screen by means of two rectangles filled with a static pattern. The rectangle corresponding to the immediate option was occluded by black lines with orientation, thickness, and spacing identical to the lines that would occlude the photograph at the time of reward (see Fig 2). The rectangle corresponding to the delayed option was not occluded but contained a loading bar depicting the delay before the full photograph would be visible. Decisions were self-paced. To discourage unconsidered responding, responses could only be provided 2 seconds after choice presentation, indicated by thickening of the rectangular frames. Participants responded by selecting the right or left key labeled on a computer keyboard and validated their response by pressing a central key. Following response validation, if the immediate option was chosen, the computer displayed a full screen image of the photograph occluded by black lines. If the delayed option was chosen, the delayed option was highlighted, and the loading bar progressively filled in, with duration corresponding to the prescribed delay. The full view of the photograph was then displayed. Photographs were displayed for 5 seconds and were followed by presentation of a fixation point for 0.5 second prior to onset of the next trial.

Participants were given detailed instructions at the beginning of the task and a chance to practice. They were told that the study examined how people make choices about viewing pictures and how seeing a partial view of a picture may affect those choices. To highlight the quality of the pictures and promote interest, participants were told that the photographs were taken by amateur photographers for a contest, and that their task was to indicate on each trial their choice between seeing a partial view of a photograph right away or a full view of the photograph after some delay. Participants initially became acquainted with viewing occluded photographs through presentation of two successive screens, each featuring the same picture with three different occlusion levels. They were then guided through two example trials with the examiner demonstrating use of the response buttons. The same trial was presented twice so that the examiner could demonstrate the effect of choosing the occluded photograph immediately as well as the full view photograph after the specified delay. Participants then completed 8 practice trials on their own. The occlusion levels and delays for the example and practice trials were predetermined but the photographs that served as reward were randomly selected from the aforementioned bank of pictures.

To promote participant engagement, following every five trials a screen appeared asking participants to rate their subjective experience during the last viewed photograph using a Likert scale ranging from extremely unpleasant (-4) to extremely pleasant (+4). After a rating was entered, a screen labeled “Processing Data” appeared, with a screen-wide loading bar that progressively filled in. The wait time during that screen was designed to equalize experimental time across participants: it lasted five seconds plus the duration of all non-chosen delayed options during the previous five trials. Participants were not informed of the rationale for this “Processing Data” screen, but were told during instructions that their decisions would not impact the duration of the task or the number of photographs that they would view. This procedure insured that choosing the immediate viewing option would not result in seeing more pictures or completing the task sooner, an experimental confound that has been related to increased discounting in humans [82]. Task duration adjustment was carried out every five trials rather than every trial to prevent direct mental association of selecting the immediate option with subsequent waiting and potential interpretation of such waiting as punitive. Cell phone usage or other overt distraction was not permitted during these wait periods to eliminate external factors that might impact the decision process.

#### Pleasantness rating task

**Fig 3:**
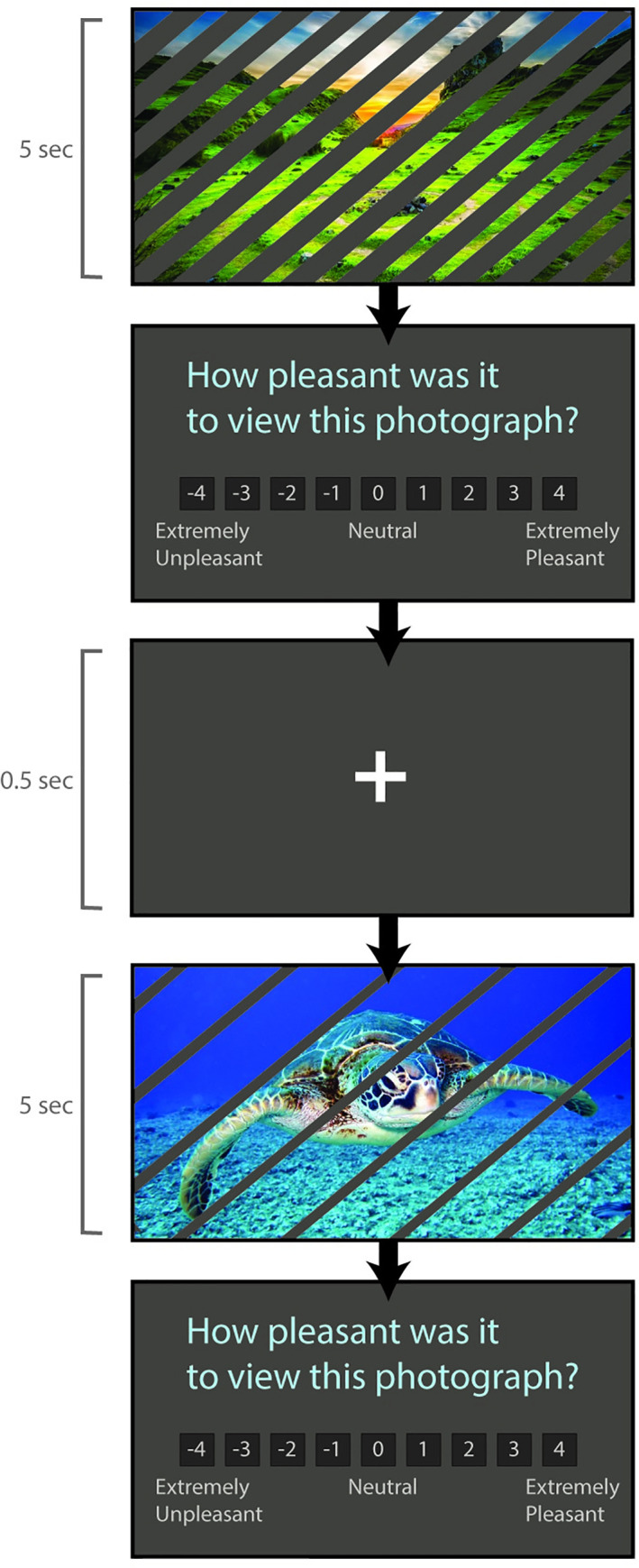
Pleasantness rating task. Participants were presented with a series of novel photographs with varying occlusion levels and asked to rate the pleasantness of their experience.

To evaluate the subjective reward value of viewing photographs occluded by lines, participants were administered a pleasantness rating task (Fig 3). Participants were presented with a series of novel photographs with varying occlusion levels, randomly selected among the bank of photographs that were not used during the experiential intertemporal choice task. After presentation of each photograph, viewed for 5 seconds, participants rated the pleasantness of their experience using a Likert scale ranging from extremely unpleasant (-4) to extremely pleasant (+4). A fixation cross presented for 0.5 second separated successive trials. Six trials were presented for each of the 10 occlusion levels, for a total of 60 rating trials.

#### Hypothetical intertemporal choice task

**Fig 4:**
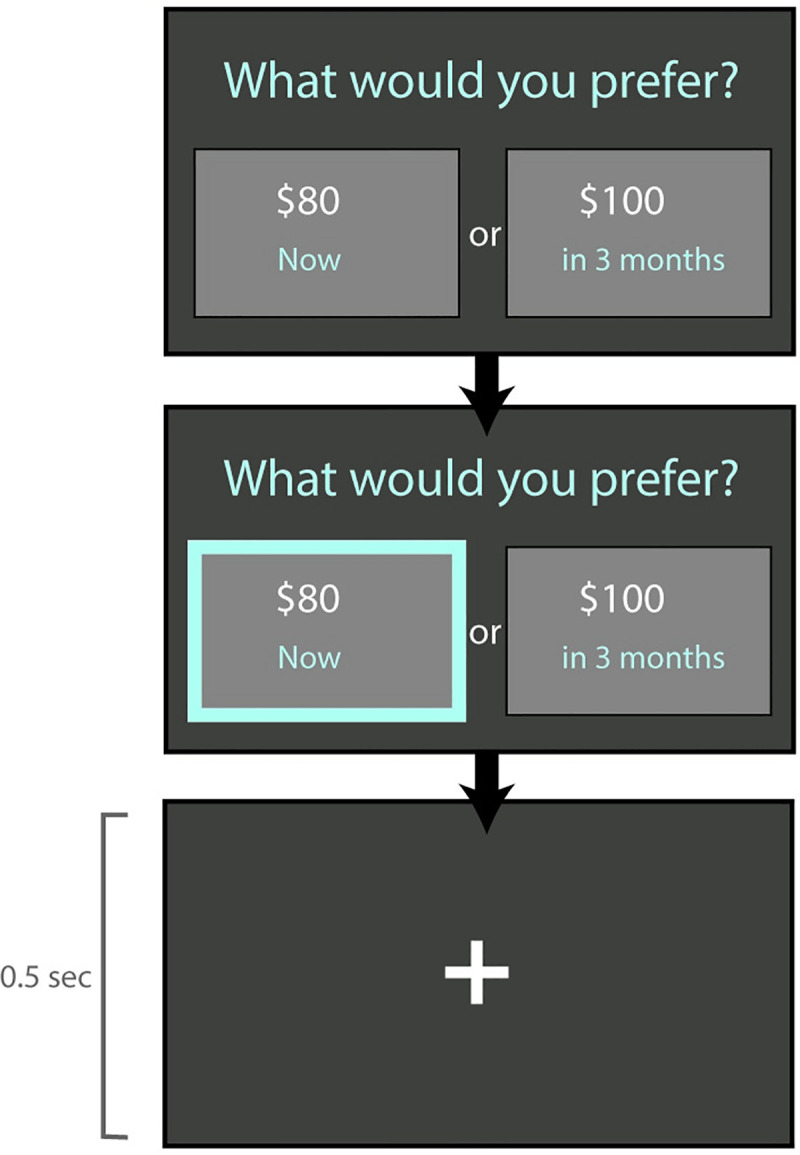
Hypothetical intertemporal choice task. The task required making a series of hypothetical decisions between receiving an amount of money varying between $1 and $99 now or receiving $100 after a delay.

The hypothetical task involved a series of hypothetical decisions between varying amount of money “Now” and $100 after varying delays (Fig 4). Possible amounts for the immediate options were: $1, $5, $10, $20, $30, $40, $50, $60, $70, $80, $90, $95, $99, and $100. Delays included 1 day, 2 days, 1 week, 2 weeks, 1 month, 3 months, 6 months, 1 year, 2 years, 5 years, and 10 years. The structure of the task was similar to the experiential task, with a 2-second buffer-time before being able to provide a response, a two-step response selection and validation process, and a semi-adaptive algorithm run twice for efficient determination of two indifference points per delay. Unlike the experiential task where the consequences of the choices were experienced, response selection was immediately followed by presentation of a fixation cross (0.5 seconds) and onset of the next trial. Based on findings of little evidence of systematic difference in temporal discounting patterns between incentivized and unincentivized tasks [27], we did not use actual money payouts.

All tasks were programmed and displayed using the Matlab^TM^ Psychtoolbox-3 [83], and administered on a L1951p 19-inch LCD monitor with 1440 x 900 resolution. The order of intertemporal choice tasks was counterbalanced across participants, with the pleasantness rating task always performed immediately following the experiential intertemporal choice task.

#### Measures of state and trait anxiety

State and trait anxiety were measured using the State Trait Anxiety Inventory [84]. This instrument is a self-report questionnaire divided into a state anxiety subscale, which assesses anxiety symptoms experienced by the participant in the moment, and a trait anxiety subscale, which assesses frequency of anxiety symptoms generally experienced by the participant. Each scale comprises 20 items, rated on a 1 to 4 scale. The inventory was administered before the experimental tasks.

### Analytic approach

#### Hyperbolic temporal discounting

To evaluate whether the experiential intertemporal choice task yielded valid delay-reward tradeoff, pairs of indifference points obtained for each delay and each participant were fit to a model of reward subjective values (SV) decreasing hyperbolically with increasing delay [85]:

$$SV=\frac{M}{1+k\times T},$$ (1)

where *M* is the objective value of the reward, *k* is the discounting rate, and *T* is the delay. Because the value of the delayed reward was kept constant, that value was normalized to *M = 1*. Minimum subjective values were *SV*_*min*_ *= $1* in the hypothetical task and *SV*_*min*_ *=* 13% of visible photograph in the experiential task. The SVs were transformed to set the minimum subjective value to 0, as assumed in the hyperbolic model:

$$SV^{\prime }=\left(SV-{SV}_{min}\right)/\left(1-{SV}_{min}\right).$$ (2)

Model assessment was carried out by means of two complementary analyses: the first evaluated amount of tradeoff between delay and reward in each individual separately through calculation of *R*^*2*^, and the second evaluated model fit using linear mixed modeling. For the first analysis, best fit hyperbolic curves were first calculated for each participant using least square fit, implemented using the *fmincon* minimization function of Matlab^TM^. *R*^*2*^ values were then computed, that quantify the proportion of variance additionally explained by the model compared to the mean of the participant’s indifference points:

$$R^{2}=1-{SS}_{R}/{SS}_{Tot},$$ (3)

where *SS*_*R*_ represents the sum of squares of the model’s residuals and *SS*_*Tot*_ represents the variance about the data’s mean. Negative *R*^*2*^ values suggest that the data are better described by the data’s mean. Consistent with previous work [63], we report median values as well as the proportion of individuals for whom the model does not provide better description of the data than the mean. A paired Wilcoxon Signed Rank Test was used to compare median *R*^*2*^ values across the experiential and hypothetical tasks.

For the evaluation of model fit (i.e., how close the model is to the data points), using *R*^2^ is not appropriate as it confounds model fit with discounting rate [86]. That is, the more a participant is willing to wait and thus the “flatter” their indifference point curve, the worse the *R*^2^ value. Hyperbolic fit was evaluated instead using linear mixed modeling with participant as a random factor. This analysis allows fitting the indifference data points of all participants at once, thus simultaneously taking into account within and across subject variance. It also provides a test of significance of the hyperbolic model fit compared to the null model defined by separate means for each participant that takes into account the different number of parameters across models. The model was constructed using algebraic reformulation of Eq (1) into a linear form. (The log-form was chosen for purpose of comparison with the logistic model proposed in the next section.)

log((1−SV′)/SV′)=logk+logT. (4)

The linear mixed model was defined as:

$$Y_{ij}=\beta_{0i}+\log T_{j},$$ (5)

where *Y*_*ij*_ represents the transformation of the subjective value ${SV}_{ij}^{\prime }$ for the *i*^th^ participant and *j*^th^ delay, such that $Y_{ij}=\log\left(\left(1-{SV}_{ij}^{\prime }\right)/{SV}_{ij}^{\prime }\right)$, and where log *T*_*j*_ is the log-transform of the *j*^th^ delay. (Log base e is assumed throughout the paper). The model implicitly includes the discounting rates log *k* as intercepts (*β*_0*i*_), determined for every participant (random effect) and on average for the group (fixed effect). The regression coefficient associated with log *T*_*j*_ was fixed to *1*. Model fit was carried out using maximum likelihood as implemented in the *lme4* package [87] of R [88]. The model was compared to a null model defined by the mean of the indifference points data, constant with respect to delay, but allowed to vary across participants: *Y*_*ij*_ = *β*_0*i*_. Model fit was evaluated using the Akaike’s Information Criterion (AIC) [89] and Bayesian Information Criterion (BIC) [90]. *Rsquared* effect size was estimated using the method developed by Nakagawa & Schielzeth [91], implemented with the *piecewiseSEM* R package. Model comparison was carried out using a Likelihood Ratio Test with χ^2^-distribution. Goodness of fit was also evaluated for each participant separately from the least square fit results by calculating the root mean square error (*RMSE*), which quantifies the average distance between the model and the data independently from the data’s mean:

$$RMSE=\sqrt{{SS}_{R}/n},$$ (6)

where n is the number of data point (see [92] for an example of use of *RMSE* in the context of temporal discounting).

#### A logistic function framework for temporal discounting

Evidence suggests that a simple hyperbolic model may not provide optimal fit of temporal discounting data [62, 63]. A logistic function [64] framework is proposed here:

$$SV=\frac{1}{1+e^{a(\log T-b)}},$$ (7)

where log *T* is the log-transform of the delay, *b* the log-delay at inflection point, and *a* the slope at inflection point. This model was developed by noting that the hyperbolic curve can be expressed as a one-parameter logistic function, with slope at inflection point assumed to be fixed to *a = 1*, and with log-delay at inflection point characterized by the classic discounting rate: *b* = −log *k* (see Fig 1). As mentioned in the introduction, the logistic function is proposed as a more intuitive framework to interpret indifference point curves, but this model is not new mathematically and, in another form, has been demonstrated to provide superior fit compared to classic models of temporal discounting [63, 66, 67].

Again, two methods were used for assessment of model fit, the first involving least square fit on indifference points data to assess delay-reward tradeoff in each participant, and the second involving linear mixed modeling to assess goodness of fit of the model on all the participants’ indifference point data simultaneously. The linear mixed model was developed using reformulation of Eq (7) into the following linear form:

log((1−SV′)/SV′)=−a×b+a×logT. (8)

The model was then expressed similarly to Eq (5), but with an additional regression parameter:

$$Y_{ij}=\beta_{0i}+\beta_{1i}\log T_{j}.$$ (9)

The model implicitly includes the negative product of parameters*–a × b* as the intercept *β*_0*i*_ and parameter *a* as the regression coefficient *β*_1*i*_. Both were determined for each participant separately (random effects) and on average for the group (fixed effects). The logistic model was tested on experiential and hypothetical indifference point data and compared to the hyperbolic model using the AIC and BIC indices of fit, Rsquared effect size, and Likelihood Ratio Test. Again, the likelihood ratio test provided a test of significance that permitted comparison of model fit, taking into account the different number of parameters across models. *RMSE* was also calculated for each participant separately for an indication of individual goodness of fit.

#### Perceptual reward sensitivity

To examine the association between level of occlusion of the photographs and experienced reward in the pleasantness rating task, linear mixed modeling was carried out with participant as random factor. The model was defined as follows:

$${Pleasantness}_{ij}=\beta_{0i}+\beta_{1i}{Occlusion}_{j},$$ (10)

where *Pleasantness*_*ij*_ represents the rating of pleasantness experienced by participant *i* after viewing a partially occluded photograph *j*, *Occlusion*_*j*_ is the level of occlusion of that photograph, and regression coefficient *β*_1*i*_ is included both as fixed and random effects. The model also included fixed and random intercepts, *β*_0*i*_. Model fit was tested against a null model, where pleasantness ratings could be different on average for each participant, but remained constant as a function of occlusion: *Pleasantness*_*ij*_ = *β*_0*i*_. Model comparison was carried out using the AIC and BIC indices of fit, Rsquared effect size, and Likelihood Ratio Test. Pearson’s product moment correlations between pleasantness ratings and occlusion levels were also calculated for each participant, with absolute values used as input in subsequent analyses to model individual differences in *reward sensitivity* when viewing partially occluded photographs.

We also examined the relationship between levels of occlusion and pleasantness ratings given following every five trials during the intertemporal choice task. This analysis was limited by the occlusion levels chosen by participants but can arguably provide more direct insight into participants’ reward experience while making intertemporal decisions. We used the same linear mixed model as Eq (10), but because the range of occlusion levels was limited by participant choice, included occlusion level only as a fixed factor. The limited range of occlusion levels did not permit individual assessment of reward sensitivity based on these ratings.

#### Model-based area under the curve

To evaluate the relation between experiential and hypothetical temporal discounting, we first computed measures of discounting for each participant and each task using area under the indifference points curve, *AUC* [93], where greater *AUC* indicates greater willingness to wait. We used *AUC* rather than the discounting parameter *k*, because it captures the entire shape of the indifferent point curve. *AUC* is usually calculated by drawing trapezes between indifference points and the x-axis, and by summing the trapeze areas together. However, because this model-free technique can be disproportionately affected by noise in the data, we opted to derive a model-based *AUC* by carrying out a preliminary fit of the data with the two-parameter logistic model described above (using the least-square fit results), and by integrating this curve over the delay interval. The relation between experiential *AUC* and hypothetical *AUC* was tested using Pearson’s product moment correlation. The Bayes factor test for linear correlation was also carried out to quantify evidence in favor of the null hypothesis. This test was implemented with the R *BayesFactor* package [94], using a single chain with 10,000 iterations, no thinning, and Markov Chain Monte Carlo sampling.

#### Effects of state and trait anxiety

The effects of state and trait anxiety on performance in experiential and hypothetical tasks were simultaneously examined using path analysis. A first path analysis used state and trait anxiety as predictors of experiential *AUC* and hypothetical *AUC*. The second analysis used the same model with the addition of *reward sensitivity* (i.e., the strength of the relation between *Occlusion* and pleasantness ratings) as possible mediator for the effect of state anxiety on experiential *AUC*. Both models included parameters for the variances of state anxiety and trait anxiety, covariance of state anxiety with trait anxiety, residual variances of experiential *AUC* and hypothetical *AUC*, and residual covariance of experiential *AUC* with hypothetical *AUC*. Parameters were estimated using maximum likelihood with the R *lavaan* package [95]. Standardized parameters were calculated with respect to the variance of state anxiety and trait anxiety. The fit of the model was evaluated using the Comparative Fit Index, CFI [96], Root Mean Square Error of Approximation, RMSEA [97], and Standardized Root Mean Square Residual, SRMR [98].

## Results

### Validating the experiential temporal discounting task

#### Hyperbolic fit

Least square fit carried out using the hyperbolic model on the experiential indifference points of each participant yielded mean average residual error *RMSE* = 0.131 (SD = 0.080). Median *R*^*2*^, representing the amount of variance accounted for compared to the mean of the indifference points was 0.520. For 7 out of 44 participants (15.9%) the hyperbolic model did not explain more variance than the mean of the participant’s indifference points. Examination of individual indifference point data suggested exclusive preference of the delayed reward in 3 participants, generally consistent choice of the delayed option with some variability in 3 participants, and seemingly random responding in 1 participant. Linear mixed modeling using the hyperbolic model on all participants simultaneously revealed a model fit (*AIC* = 6306, *BIC* = 6320, *Rsquared* = 0.44) that was better than that of the null model (*AIC* = 6483, *BIC* = 6498, *Rsquared* = 0.39), with difference in fit quality that was significant (*χ*^*2*^*(0)* = 177.2, *p* < .001). The intercept in the hyperbolic model, corresponding to the average log-discounting rate *log k*, was estimated to be *β*_*0*_ = -6.7 (standard error *SE* = 0.8). Taken together, these results validate the presence of a delay-reward tradeoff for most participants during the experiential intertemporal choice task, with the hyperbolic shape providing better approximation than the mean of the indifference points.

Least square fit carried out using the logistic function model on the hypothetical indifference points yielded mean average residual error *RMSE* = 0.132 (SD = 0.041) and median *R*^*2*^ = 0.806. For one participant the mean provided a better fit. For reference, McKerchar and colleagues [63] reported a median *R*^*2*^ of 0.929 for the hyperbolic model and found that the mean provided a better fit for 10 out of 64 individuals (16%). Linear mixed modeling further confirmed that the fit with the hyperbolic model (*AIC* = 5708, *BIC* = 5723, *Rsquared* = 0.28) was better than that with the null model (*AIC* = 6341, *BIC* = 6355, *Rsquared* = 0.16), with a difference in fit quality that was significant (*χ*^*2*^*(0)* = 632.3, p < .001). The intercept in the hyperbolic model was estimated to be *β*_*0*_ = -9.0 (*SE* = 0.4).

A summary of the fit results is presented in Table 1. Comparison of indices across the experiential and hypothetical intertemporal choice tasks suggested equivalent fit in terms of *RMSE* (*t(43)* = 0.136, *p* = 0.892, *Cohen’s d* = 0.021), but less variance explained compared to the mean in the experiential data (paired Wilcoxon Signed Rank Test on *R*^*2*^ values: *V* = 112, *p* < .001). This difference reflects the larger proportion of individuals who displayed consistent to almost consistent choice of the delayed option in the experiential task (poor *R*^*2*^ but good to excellent fit).

**Table 1:** Goodness of fit of the experiential and hypothetical indifference points, comparing fit by the hyperbolic model, logistic function model, and null model (defined as the mean of the indifference points for each participant).

		EXPERIENTIAL TASK			HYPOTHETICAL TASK		
		Null	Hyperbolic	Logistic	Null	Hyperbolic	Logistic
*Least Square Fit*	*Mean RMSE* (SD)	0.193 (0.112)	0.131 (0.080)	0.102 (0.067)	0.290 (0.087)	0.132 (0.041)	0.114 (0.041)
	*Median R*^*2*^	-	0.520	0.657	-	0.806	0.850
*Linear Mixed Modeling*	*AIC*	6483	6306	5817	6341	5708	5384
	*BIC*	6498	6320	5847	6355	5723	5413
	*Rsquared*	0.39	0.44	0.81	0.16	0.28	0.83

#### Logistic function fit

Least square fit carried out using the logistic model on the experiential indifference points of each participant yielded mean average residual error *RMSE* = 0.102 (SD = 0.067) and median *R*^*2*^ = 0.657 (see Table 1 for a summary). For 9 out of 44 participants (20.5%) the logistic model did not explain more variance than the mean of the participant’s indifference points. These individuals were the same as those described in the previous section, plus two individuals whose profiles suggested onset of delay-reward tradeoff at the tail of the delay interval. Excluding these individuals did not alter the pattern of findings described below. Linear mixed modeling using the logistic function model on the experiential intertemporal choice data revealed a model fit for all participants taken together (*AIC* = 5817, *BIC* = 5847, *Rsquared* = 0.81) that was significantly better than the hyperbolic model (*χ*^*2*^*(3)* = 494.5, *p* < .001). The estimates of the fixed effect regression coefficients were *β*_*0*_ = -13.5 *(SE = 0*.*7)* and *β*_*1*_ = 4.3 *(SE = 0*.*4)*, the latter corresponding to the average estimate of parameter *a*.

Least square fit carried out using the logistic function model on the hypothetical indifference points yielded mean average residual error *RMSE* = 0.114 (SD = 0.041) and median *R*^*2*^ = 0.850. There was no participant for whom the logistic function did not explain more variance than the mean of the participant’s indifference points. For reference, McKerchar and colleagues [63] reported a median *R*^*2*^ of 0.963 for the Rachlin’s power function model, a model equivalent to the logistic function model, and had no individual for whom the mean provided a better fit. Linear mixed modeling revealed model fit using the logistic function model (*AIC* = 5384, *BIC* = 5413, *Rsquared* = 0.83) that was significantly better than that with the hyperbolic model (*χ*^*2*^*(3)* = 330.6, p < .001). The estimates of the fixed effect regression coefficients were *β*_*0*_ = -12.4 *(SE = 0*.*6)* and *β*_*1*_ = 1.8 *(SE = 0*.*1)*.

**Fig 5:**
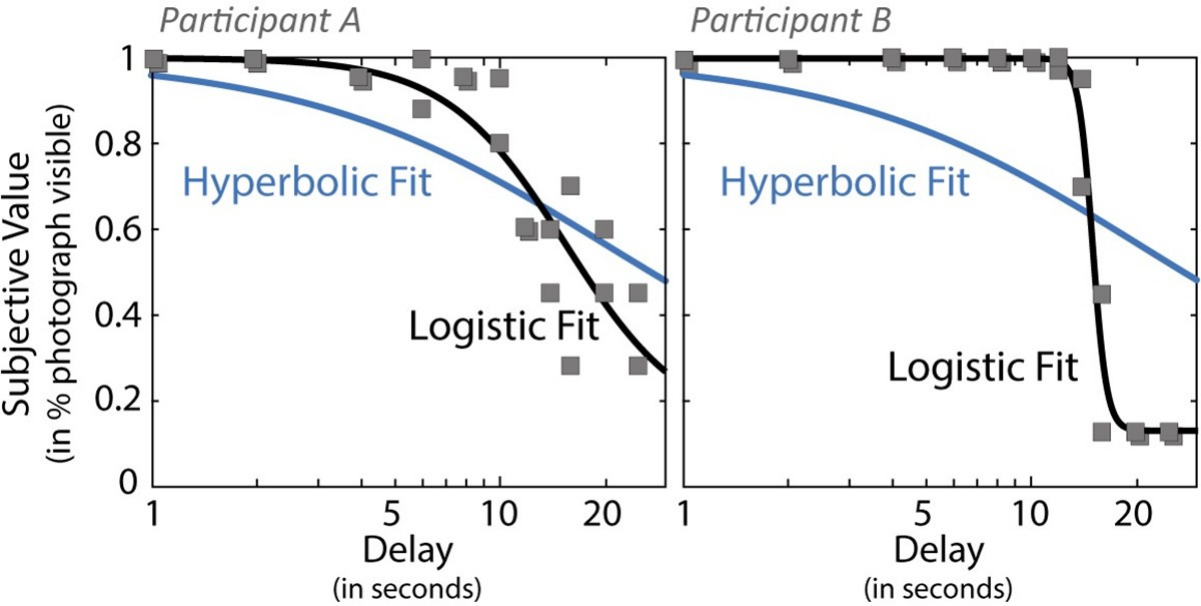
Fitting indifference point data. Example of indifference points obtained for two participants during the experiential intertemporal choice task (squared markers), and comparison of fit using a logistic function (black solid line) and a hyperbolic function (blue solid line). Delay is presented using a logarithmic scale.

**Fig 6:**
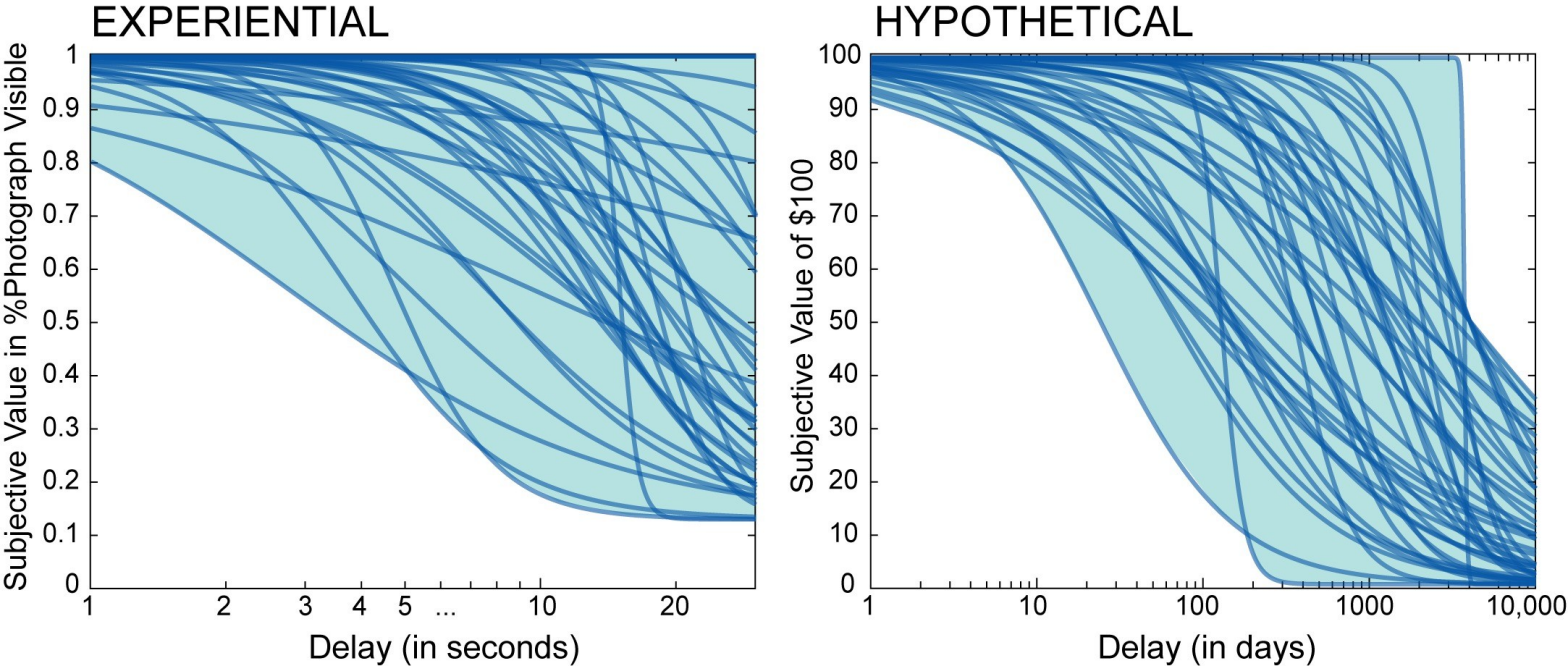
Logistic temporal discounting curves. Logistic temporal discounting curves resulting from fitting indifference point data for the experiential (left) and hypothetical (right) intertemporal choice tasks. Temporal discounting curves are depicted for each participant and each task. Delays are presented using logarithmic scales.

Example indifference point datasets obtained for two participants during the experiential intertemporal choice task are presented in Fig 5, together with results of the logistic and hyperbolic fits. The figure illustrates the flexibility of the logistic model with its variable slope compared to the hyperbolic model. Best fit logistic curves obtained for each participant using least square fit are presented in Fig 6 for the experiential and hypothetical intertemporal choice tasks. Comparison of indices across the experiential and hypothetical intertemporal choice tasks suggested equivalent model fit in terms of *RMSE* (*t(43)* = 1.031, *p* = 0.308, *Cohen’s d* = 0.155), but less variance explained compared to the mean in the experiential data (paired Wilcoxon Signed Rank Test on *R*^*2*^ values: *V* = 161, *p* < .001). This difference again reflects the larger proportion of individuals who displayed consistent to almost consistent choice of the delayed option in the experiential task (poor *R*^*2*^ but good to excellent fit).

#### Verifying perceptual reward sensitivity

**Fig 7:**
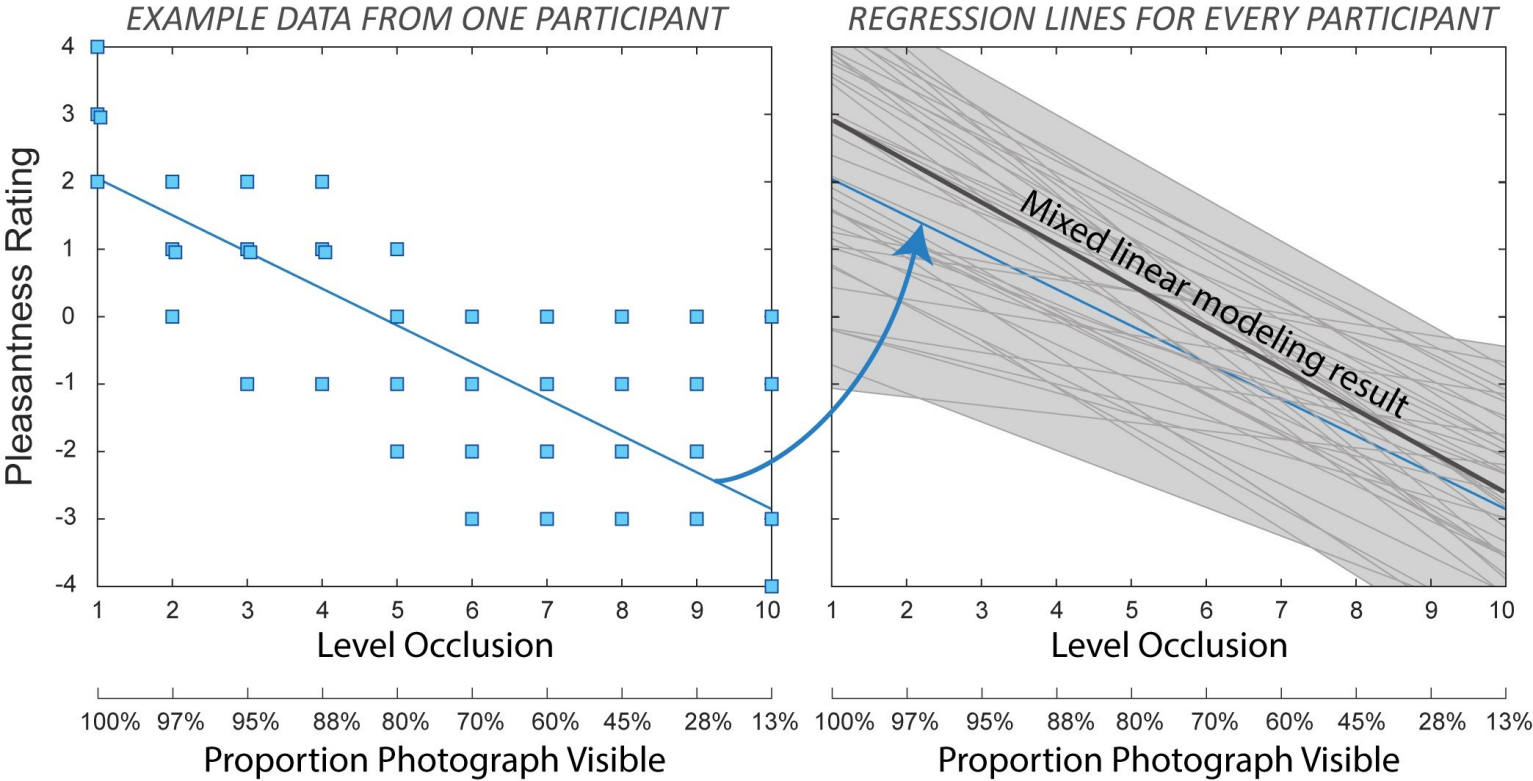
Relation between pleasantness ratings and level of occlusion of the photographs. An example of a dataset obtained for one participant is presented in the left panel. Regression lines obtained for all participants are presented in the right panel. Absolute values of correlations between pleasantness ratings and occlusion levels are used as input in subsequent analyses to represent individual differences in reward sensitivity.

The relation between photograph occlusion level and pleasantness ratings is illustrated in Fig 7, including an example dataset for one participant (left panel), and regression lines for all participants (right panel). Linear mixed modeling revealed a negative fixed effect of occlusion on pleasantness ratings (*β* = -0.55, *t (44*.*1)* = 18.1, *p* < .001). The fit of the model with occlusion levels as fixed and random effects (*AIC* = 9434, *BIC* = 9469, *R*^*2*^ = 0.84) was better than that of the null model (*AIC* = 11649, *BIC* = 11667, *R*^*2*^ = 0.19), and the difference in model fits was significant (*χ*^*2*^*(3)* = 2221, *p* < .001). Pearson’s product moment correlations calculated separately for each participant averaged *r* = -0.73 (*SD* = 0.16) and were significant (*p* < 0.05) for all but one participant. The absolute values of the correlations between photograph occlusion and pleasantness ratings were used to model individual differences in reward sensitivity in the path analyses.

Analysis of the pleasantness ratings collected during the intertemporal decision task also demonstrated a significant negative effect of occlusion level (*β* = -0.39, *t (554*.*9)* = 16.0, *p* < .001). The fit of the linear mixed model with occlusion levels as fixed effect (*AIC* = 2192, *BIC* = 2209, *R*^*2*^ = 0.41) was better than the null model (*AIC* = 2403, *BIC* = 2416, *R*^*2*^ = 0.15) and the difference in model fits was significant (*χ*^*2*^*(1)* = 213.0, *p* < .001). These findings suggest that participants as a group paid attention to the photographs and experienced them as more pleasant when they were less occluded.

### Comparing experiential and hypothetical temporal discounting

**Fig 8:**
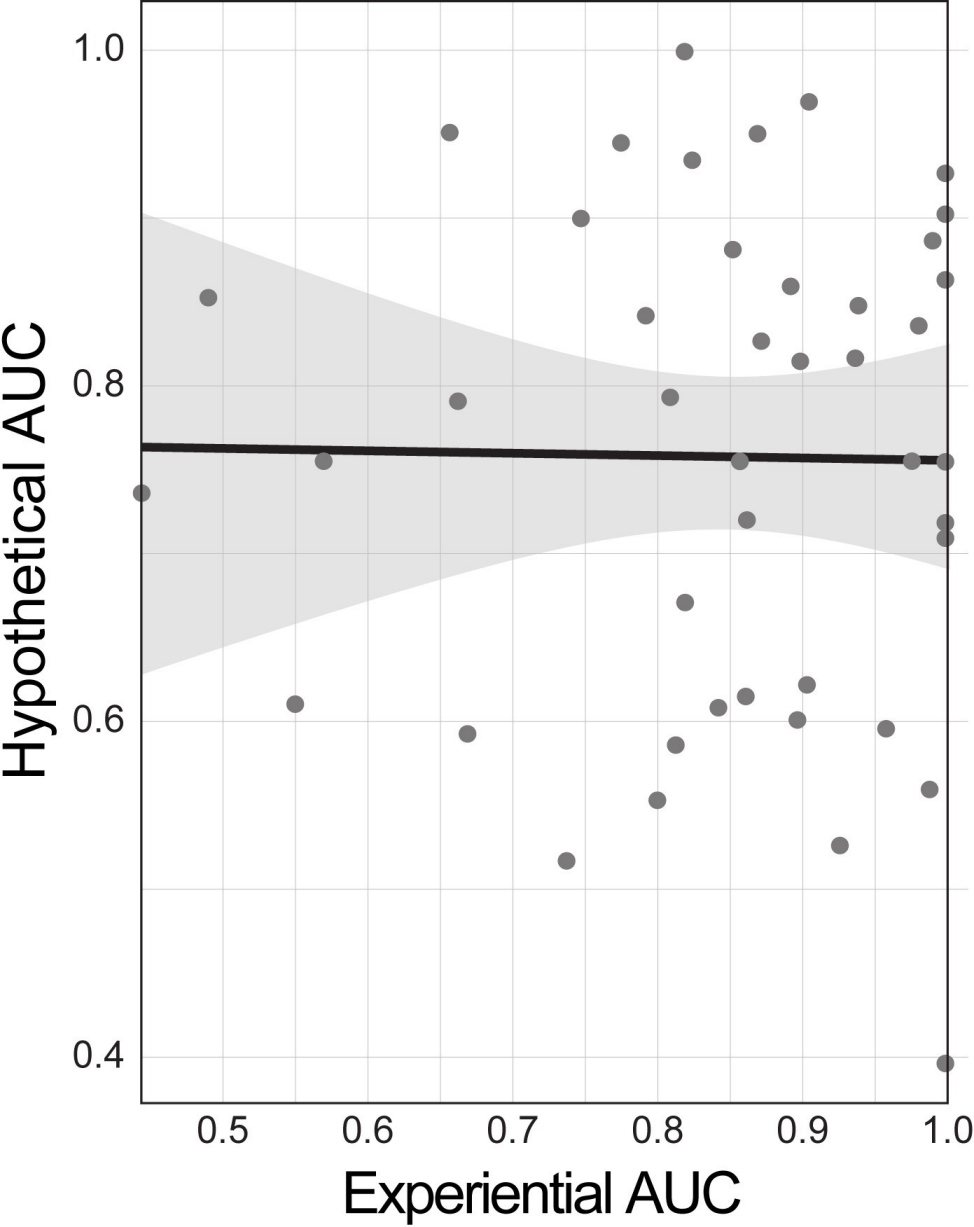
Absence of significant correlation between experiential *AUC* and hypothetical *AUC*. *AUC* stands for Area Under the Curve, with greater *AUC* implying greater willingness to wait for the delayed reward.

The Pearson product moment correlation between experiential *AUC* and hypothetical AUC was not significant (*r* = -0.014, *p* = 0.929, see Fig 8). Bayes factor test for linear correlation suggested that the data were about 3 times more likely under the null hypothesis (*r*_*median*_ = -0.013, *BF* = 0.339).

**Fig 9:**
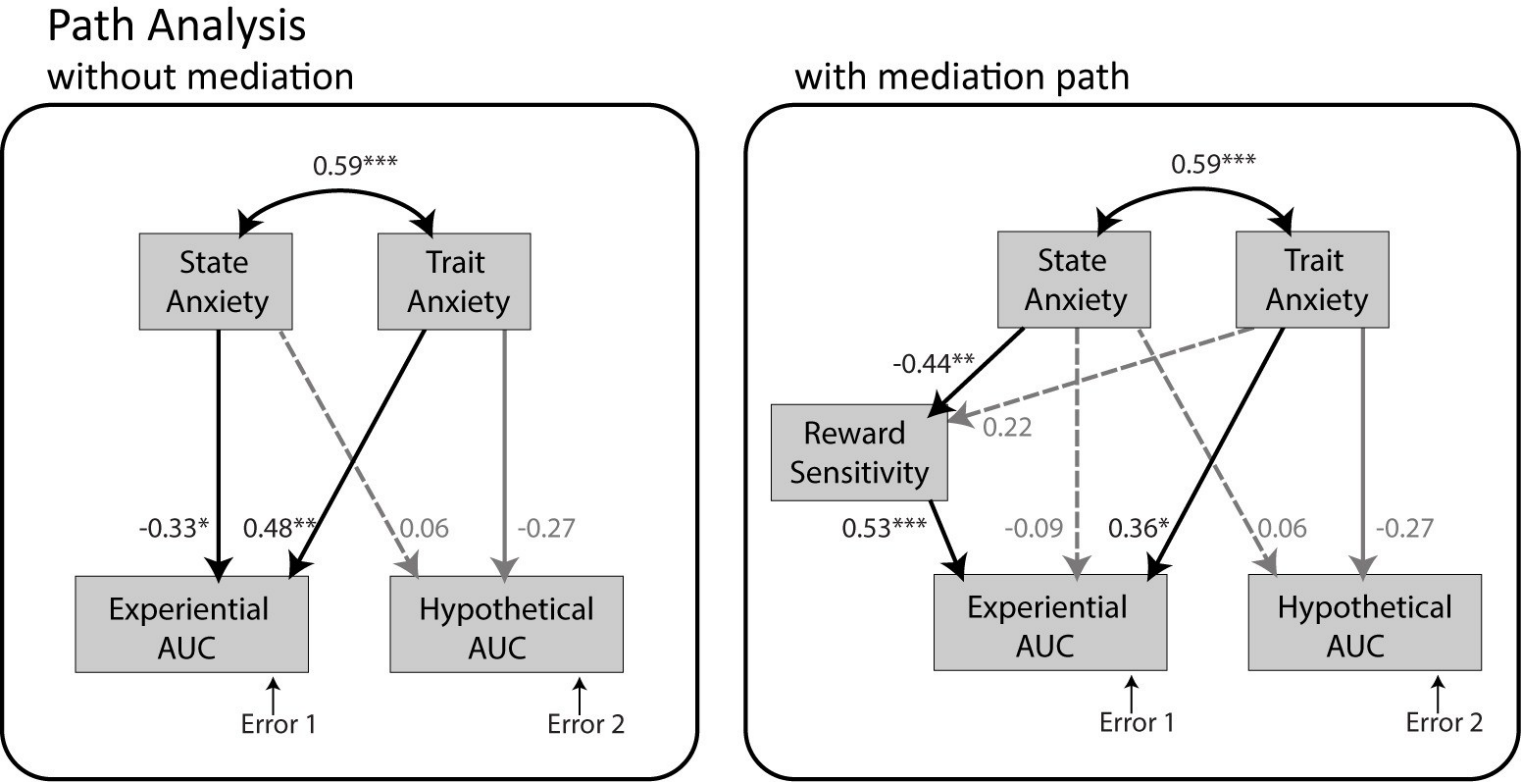
**Results of the path analysis without mediation (left panel) and path analysis with mediation (right panel).** *AUC* stands for Area Under the Curve, with greater *AUC* implying greater willingness to wait for the delayed reward. Parameter estimates are standardized with respect to the variance of state anxiety and trait anxiety. Significance level is indicated by number of stars (p < .05*, p < .01**, p < .001***).

**Fig 10:**
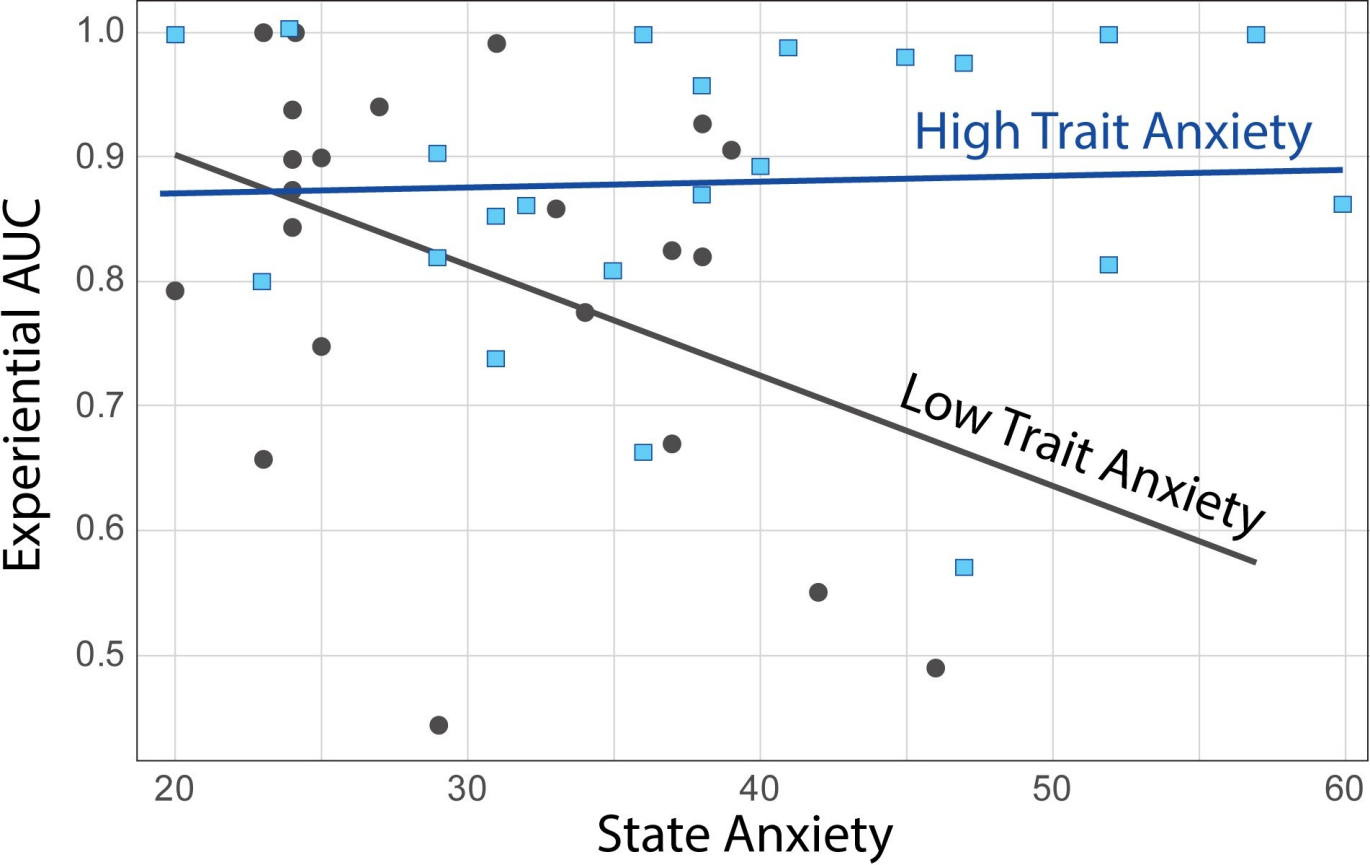
Illustration of the interaction between state anxiety and trait anxiety on *AUC* during the experiential intertemporal choice task. A split-half method was employed over the trait anxiety distribution. *AUC* stands for Area Under the Curve, with greater *AUC* implying greater willingness to wait for the delayed reward. Solid black circles correspond to data for individuals with lower trait anxiety; and blue squares to data for individuals with higher trait anxiety.

The path analysis model without mediation (Fig 9, left panel) provided a good fit of the data (*CFI  * =  1.0, *RMSEA* < .001, *SRMR* = .021). State anxiety and trait anxiety were significantly correlated (*r* = 0.59, *p* < .001). Participants reported slightly less state anxiety (*M* = 34.3, *SD* = 10.1, *range* = [20, 60]) than a normative sample of female college students [84] (*N* = 481, *M* = 38.8, *SD* = 12.0; *t(54)* = -2.78, *p* = 0.007, *Cohen’s d* = 0.406) and reported levels of trait anxiety (*M* = 43.0, *SD* = 10.8, *range* = [25, 66]) that were comparable to that normative sample (*N* = 531, *M* = 40.4, *SD* = 10.2; *t(49)* = 1.54, *p* = 0.130, *Cohen’s d* = 0.248). State and trait anxiety displayed distinct relations to experiential and hypothetical *AUC*, where greater *AUC* implies greater willingness to wait. State anxiety had a negative effect on experiential *AUC* (standardized estimate: *β*  =  -0.33, *p* = 0.049) but no significant effect on hypothetical *AUC* (*β*  =  0.06, *p* = 0.749). Trait anxiety had a positive effect on experiential *AUC* (*β*  = 0.48, *p* = 0.003). Its effect on hypothetical *AUC* did not reach significance but was noted to be numerically negative (*β*  =  -0.27, *p* = 0.124). Explanatory variables accounted for 15.0% of the variance in experiential *AUC* and 5.9% of the variance in hypothetical *AUC*. Of note, the correlations of state anxiety with experiential *AUC* (*r* = -0.05, *p* = .751) and trait anxiety with experiential *AUC* (*r* = 0.28, *p* = .063) were not significant when considered in isolation. These results highlight the presence of an interaction between state and trait factors, which is accounted for by the path analysis. Linear regression on experiential *AUC* confirmed significance of a model including state anxiety, trait anxiety, and their interactions as regressors (*F(3*,*40)* = 4.8, *p* = 0.006, R^2^ = 0.26), with a significant state by trait anxiety interaction (*β* = .0005, *t* = 2.5, *p* = 0.017). This interaction is illustrated in Fig 10 using a split-half method: individuals with lower trait anxiety generally displayed experiential *AUC* that decreased (less willingness to wait) with increasing state anxiety (*r* = -0.41, *p* = .057); while individuals with higher trait anxiety displayed experiential *AUC* that remained high, independent of their state anxiety (*r* = 0.04, *p* = .850).

The path analysis model with mediation (Fig 9, right panel) provided a good fit of the data (*CFI * =  1.0, *RMSEA* < .001, *SRMR* = .018). State anxiety had a negative effect on reward sensitivity (*β*  =  -0.44, *p* = .007), which itself had a positive effect on experiential *AUC* (*β*  =  0.53, *p* < .001). The direct effect of state anxiety on experiential *AUC* was no longer significant (*β*  =  -0.09, *p* = .543), suggesting complete mediation through reward sensitivity. The effect of state anxiety on hypothetical *AUC* remained the same, and was non-significant (*β*  =  -0.06, *p* = .749). Trait anxiety had a non-significant effect on reward sensitivity (*β*  =  0.22, *p* = .198), but still had a positive effect on experiential *AUC* (*β*  =  0.36, *p* = .013). Its non-significant effect on hypothetical *AUC* remained the same (*β*  =  -0.27, *p* = .124). Explanatory variables accounted for 12.9% of the variance in reward sensitivity, 39.3% of the variance in experiential *AUC* and 5.9% of the variance in hypothetical *AUC*.

## Discussion

The present study explored the involvement of distinct processes underlying experiential and hypothetical intertemporal choices. First, a novel experiential task and proposed logistic function interpretive framework were validated. Second, comparison of performance across experiential and hypothetical choices revealed that temporal discounting across the two tasks was not related and had different patterns of relations to trait and state measures of anxiety.

### A novel experiential task and a logistic function framework to examine temporal discounting

The current study used a novel experiential intertemporal choice task with delays of seconds experienced in the moment and partially occluded artistic photographs as consumable rewards. We observed a significant association between photograph occlusion and pleasantness ratings, suggesting that the stimuli elicited a continuum of reward values for the participants. Further, we demonstrated the presence of an effective tradeoff between delay and reward, indicating that the task elicited temporal discounting in a majority of participants. The present task built on previous studies that have used pleasant pictures as reward [11, 55], but rather than using duration of reward exposure to manipulate reward magnitude, we manipulated how much of the information contained in the pictures would be available. As such, the current task permits separating the time processes involved in the experience of the delay from those involved in the experience of the reward. In addition, compared to previous studies that relied on social or sexual attraction, the current task taps into the rewarding effect of novel perceptual information more generally [59], and can thus be applied in future studies to a variety of populations including children. Moreover, an easily implemented experiential task may be particularly useful for the study of maladaptive behaviors such as substance dependence, cigarette smoking, or overeating, given that actual consumption of rewards is inherent to difficulties with intertemporal decisions in such situations.

A logistic function interpretive framework was also proposed, providing superior fit of temporal discounting indifference points compared to the classic hyperbolic model [1, 61]. The logistic function framework was developed through mathematical inference, by noting that the hyperbolic model constitutes a subset of the logistic function with logarithmic timescale and constant slope at the inflection point. Using the logistic function framework has several advantages. First, using a logarithmic time scale in intertemporal choice accounts for the diminished perception of a change in delay as the overall magnitude of the delay increases, as suggested by the Weber-Fechner law [65]. It also reveals the underlying presence of an S-shape in the indifference curve, and explicates the negative logarithm of the classic hyperbolic discounting rate, −log *k*, as the log-delay at which a participant switches from consistently choosing the delayed to consistently choosing the more immediate option. Such a framework can be especially useful for adaptive testing where the goal is to identify the most informative part of the curve [99]. Further, the logistic function framework allows for a flexible slope at the inflection point with the incorporation of an additional parameter, *a*. Our finding that the logistic model provides a better fit than the hyperbolic model, which implicitly assumes that all discounting curves have the same slope, *a* = 1, is consistent with prior demonstrations of superior fit of a mathematically equivalent model [63, 66, 67]. This model, expressed as $SV=\frac{M}{1+k\times T^{s}}$, is identical to a logistic function model with slope *a = s* and log-delay at inflection point *b* = −log (*k*)/*s*. Consistent with Stevens’ psychophysical power law [68], parameter *a* (or *s*) may be interpreted as variability in the subjective perception of time [66]. As suggested by our findings of different average values for *a* in the experiential compared to hypothetical intertemporal choice tasks, *a* is likely to be both individual- and task-dependent.

The possibility of adding a third logistic function parameter, *c*, characterizing the asymptote at long delays, was also entertained. Such a parameter could be interpreted as the minimum amount of reward that might appear worth obtaining for a given person. For example, if $5 is considered a negligible amount of money by an individual, they might decide that it is worth waiting even 50 years for the possibility of $100, rather than getting $5 now. In other words, their asymptote may be $5 instead of $0, as assumed by the two-parameter model. Testing of model fit revealed improvement over the two-parameter logistic model that was negligible in size and not significant (*ΔRMSE* = 0.002, *t(43)* = 1.354, *p* = 0.183). The more complex three-parameter model was thus discarded for parsimony.

### Comparing experiential and hypothetical intertemporal choice

#### Temporal discounting across tasks

Comparison across experiential and hypothetical choice tasks revealed that measures of temporal discounting were not correlated, suggesting the presence of distinct underlying mechanisms. Although the possibility that a weak correlation could be detected in a larger sample of participants cannot be excluded, based on the Bayes factor test, such possibility is three times less likely than a null correlation. Moreover, based on the effect size in the current sample (i.e., r = -0.014, p = 0.929), such correlation would likely be extremely small to negligible compared to correlations between hypothetical tasks reported in previous studies (e.g., generally ranging between 0.3 and 0.9 in [29]). The lack of correlation between tasks observed here is in line with the results of Johnson [38], who similarly found that decisions across an experiential task (using coins immediately available) and a hypothetical task (using hypothetical amounts of money) were not significantly correlated. The results, however, contrast with those of Lukinova and colleagues [37] whose experiential task used rewards that could be experienced less directly (depiction of money on a computer screen). Taken together, these results may point to the consumability of rewards as an important feature of experiential tasks; that is, it may be critical that rewards are available to direct experience if they are to inform the value of subsequent choices. This notion is also consistent with findings demonstrating that intertemporal choices in animals depend more critically on the delay governing the exchange of a secondary reinforcer against food, rather than on the delay associated with presentation of that secondary reinforcer [40].

#### Effects of state and trait anxiety across tasks

Choices in the experiential and hypothetical tasks were differentially associated with state and trait anxiety. When state and trait anxiety, which were positively related, were simultaneously taken into account, state anxiety was associated with increased discounting in the experiential task but not in the hypothetical task, consistent with our prediction. The selective association of state anxiety with temporal discounting in the experiential task aligns with results of previous studies showing effects of state manipulations on choices in an experiential task but not in a classic hypothetical task [70, 71]. Supporting the notion that state anxiety impacts the *experience* of reward in the experiential task, we found that the relation of state anxiety with experiential discounting was mediated by participants’ sensitivity to the reward value of pictures with varying levels of occlusion. That is, state anxiety was associated with decreased reward sensitivity (see also [72, 73]), which in turn was associated with increased discounting. By contrast, state anxiety was not associated with hypothetical discounting, suggesting that current state is not related to the conceptual understanding of monetary value and associated decisions.

The effects of trait anxiety were more complex. Greater trait anxiety was numerically, albeit non-significantly, associated with increased discounting in the hypothetical task. This association was weaker than that seen in some prior studies [75–77] but compatible with variability in the literature [78]. Interestingly, trait anxiety had a significant and numerically opposite association with temporal discounting in the experiential task, with greater trait anxiety associated with increased willingness to wait for greater perceptual rewards. Unlike for state anxiety, this relation was not mediated by reward sensitivity, perhaps reflecting lesser involvement of in-the-moment processes. One way to understand the positive association of trait anxiety with willingness to wait in the experiential task may be with reference to the inherent conflict that underlies trait anxiety, characterized by concomitant drives for both approach and avoidance [100]. For example, social anxiety is characterized by drives for both positive social interactions and avoidance of potential humiliation [101]. Further, trait anxiety has been related to greater effort exertion associated with higher incentives [102], management of threat [103], and compensation for depleted working memory resources [104]. Thus, the experiential task, in which risk and uncertainty are minimal, may have differentially tapped the approach drive of trait anxiety and yielded increased motivation to obtain maximal rewards. By contrast, classic hypothetical intertemporal choice tasks, which involve risks and uncertainties associated with the future, have been postulated to engage the avoidance drive of trait anxiety, with reports of decreased willingness to wait associated with risk avoidance and intolerance of uncertainty [105, 106].

By taking into account both trait and state anxiety in one model, we were able to isolate their respective effects. Yet, our results also demonstrate that these variables interact, highlighting the importance of considering their joint effects on discounting. Notably, we show that the significant increase in experiential discounting observed with greater state anxiety only applied to individuals with lower trait anxiety. Individuals with higher trait anxiety appeared more willing to wait across the board, independently of their state anxiety. This pattern may suggest that trait anxious individuals are less influenced by current experience, and that instead, their choices remain governed by consideration of abstract principles. This notion is also supported by the absence of mediation by reward sensitivity of the association between trait anxiety and experiential intertemporal choice.

Interactions between state and trait anxiety have been reported in previous studies, with similar findings that state anxiety (or induced stress) yields increased discounting in individuals with low but not high trait anxiety (or trait perceived stress) [77, 107]. Of note, these studies involved hypothetical intertemporal choices, in apparent contradiction to our observation of an interaction only in the experiential task, and to state anxiety having no effect in the hypothetical task. Interestingly, in these studies, state anxiety and/or stress were induced by asking participants to imagine themselves in specific scenarios. Episodic simulation has been shown to impact hypothetical intertemporal choices, yielding increased willingness to wait with increased vividness of imagined scenarios [10, 108]. The effect of state anxiety on hypothetical intertemporal choice in these tasks might have thus been induced by episodic simulation. Consistent with this notion, a similar effect of affect manipulation on hypothetical intertemporal choice was observed to be mediated by enhanced future perspective [109].

### Cognitive and neural mechanisms supporting experiential intertemporal choice

The observed behavioral differences between hypothetical and experiential intertemporal choice may stem from differences in two distinct components of choice: valuation (the computation of subjective values of available options) or deliberation (encompassing all processes leading to a choice) [110, 111]. In contrast to the hypothetical intertemporal choice task, where the subjective values of options are known ahead of time by virtue of semantic knowledge, subjective values in the experiential task need to be computed on the fly, requiring the encoding of the aversive experience of the delay, the encoding of the pleasant experience of the reward, and their integration into a novel representation. Such construction of values de-novo has been proposed to engage the hippocampus [112], likely relying on its unique properties in complex relational encoding [113, 114]. This mechanism is further supported by the identification of a group of cells in the hippocampal CA1 subfield specializing in encoding delay duration, reward amount [115] and reward-context conjunction [116, 117].

Differences in behavior across the experiential and hypothetical tasks may also reflect differences in deliberative processes. In hypothetical intertemporal choice, because all decisions are made before consequences can be experienced, deliberation can be carried out conceptually, relying solely on semantic knowledge and reasoning. By contrast, in experiential tasks, choices may initially be based on semantic knowledge and reasoning but can be revisited based on the experience of the delays and rewards during the task. That is, participants can sample from past experience to mentally evaluate possible outcomes and weigh them against each other, a process also demonstrated to rely on the hippocampus [118–120]. This notion is consistent with findings that animals with surgical hippocampal lesions spend more time exploring intertemporal options before committing to a decision making strategy, and are less sensitive to changes in reward amounts [25]. It is also supported by the identification of CA1 cells that specialize in comparing the expected and actual values of experienced events [121].

Although hippocampal involvement has been reliably demonstrated in animals [17–19], it has yet to be demonstrated in experiential intertemporal choice in humans. Only two human imaging studies have employed an experiential intertemporal choice task with real-time delays and consumable rewards, and these studies did not observe hippocampal activation. However, one study focused on modeling subjective reward values rather than decision-related processes [11], and in the other study some rewards were experienced after intervening decisions were made, and consequently, choices on these intervening trials could not be shaped by experience of the outcome of the prior decision [52]. Thus, future studies are needed to determine whether the hippocampus plays a role in experiential intertemporal choices in humans.

### Limitations

Because of experimental constraints, the experiential and hypothetical intertemporal choice tasks necessarily differed not only in the experience of delays and rewards, but also in the time scale of the delays (seconds versus days to years), the costs incurred by the delays, and the nature of the rewards. Differences in time scale are unlikely to explain our findings, as evidence suggests that subjective value brain signals during intertemporal choice tasks adapt to the range of values employed in the experimental context [35], and correlations across tasks with distinct temporal scales have been reported with monetary rewards [37]. Differences in costs associated with the delay have been highlighted as a key confound in comparisons between experiential and hypothetical intertemporal choice tasks [122] and were also present in the current study: the experiential task required waiting for the reward without distraction (high delay cost), whereas the hypothetical task did not specify the conditions of the hypothetical delay, and therefore participants could presumably resume normal daily life during that period of time (low to no delay cost). Paglieri [122] may thus argue that a hypothetical task in which participants are asked to imagine themselves waiting for the delay without distraction would constitute a fairer comparison to an experiential task. However, by virtue of the demands on mental simulation that such an unusual situation may induce, this task would likely recruit neural processes beyond those involved in classic hypothetical intertemporal choice, as demonstrated by intertemporal choice tasks that involve episodic simulation [10, 108].

The use of consumable perceptual rewards in the experiential task compared to money in the hypothetical task may also have contributed to differences in discounting across our tasks. Within the context of hypothetical intertemporal decisions, several studies have documented steeper temporal discounting of consumable rewards (e.g., chips, candy, soda, beer) compared to money [33, 34, 39] but discounting rates typically have been found to be correlated, suggesting a common underlying process regardless of the type of reward. By contrast, the lack of correlation across our experiential and hypothetical tasks suggests the involvement of distinct processes. Given the importance for experiential intertemporal choices of a reward that can be directly experienced, ideally, future studies designed to compare experiential and hypothetical choices should employ consumable rewards that also lend themselves to hypothetical decisions. How to implement this requirement is challenging given the limited range of rewards with which participants have a history of experience that can form the basis for both repeated hypothetical and experiential decisions.

We measured state anxiety at the beginning of the session, but it is possible that changes in participants’ inner state took place over the course of the session, in part depending on their personal experience of photograph content. Exposure to pictures of nature scenes, compared to pictures of buildings or geometric shapes, has been associated with decreased hypothetical discounting [123–125]. Given that there was minimal variability in the proportion of photographs of nature scenes that participants viewed, the extent to which viewing nature scenes may have reduced anxiety or otherwise impacted discounting was likely similar for all participants. Nonetheless, it would be useful for future studies to monitor for changes in state anxiety across the course of the intertemporal choice task.

Similarly, the current study did not comprise ongoing measurement of reward consumption. The measure of reward sensitivity was indirect, based on pleasantness ratings that were subjective and measured after the intertemporal choice task. For a more direct and unbiased assessment of reward consumption, future studies may consider use of online psychophysiological measures such as eye-tracking.

Finally, participants in our study were all female college students. This sample promoted homogeneity for consideration of underlying mechanisms; however, generalizability of findings to males remains to be established. Effects of gender on hypothetical intertemporal choice have been mixed [126–128], with findings thought to be due in part to hormonal influences on neurobiological mechanisms of reward [129]. Such factors could differentially affect experiential intertemporal choices and may need to be controlled for in future studies. Further, in light of gender-specific effects of trait anxiety on decision making [130], it remains to be established whether the dissociable effects of trait anxiety on experiential versus hypothetical choices are similarly present in men.

## Conclusions

The present study employed an experiential intertemporal choice task using artistic photographs as consumable perceptual reward. To fit indifference point data, a two-parameter logistic function interpretive framework was proposed that accounts for individual variability not only in the delay at which an individual tends to switch from consistent choice of the delayed option to consistent choice of the more immediate option, but also in the slope of that switch.

Our findings provide support for the notion that experiential and hypothetical intertemporal choices are mediated by distinct mechanisms. Performance across the two tasks was not correlated, and both state and trait anxiety were significantly related to the experiential task but not to the hypothetical task. The involvement of distinct mechanisms in the experiential compared to hypothetical task aligns with neural evidence for the recruitment of the hippocampus in animal but not in classic human intertemporal choice.
